# Dysuria Associated with Non-Neoplastic Bone Hyperplasia of the Os Penis in a Pug Dog

**DOI:** 10.3390/vetsci8010006

**Published:** 2021-01-02

**Authors:** Noritaka Maeta, Takako Shimokawa Miyama, Kenji Kutara, Teppei Kanda, Ikki Mitsui, Akihiro Ohnishi, Masahiro Miyabe, Yuki Shimizu, Kaori Saeki, Yasuhiko Okamura, Kazuaki Yamazoe

**Affiliations:** Faculty of Veterinary Medicine, Okayama University of Science, 1–3 Ikoino-oka, Imabari, Ehime 794-8555, Japan; t-shimokawa@vet.ous.ac.jp (T.S.M.); k-kutara@vet.ous.ac.jp (K.K.); t-kanda@vet.ous.ac.jp (T.K.); i-mitsui@vet.ous.ac.jp (I.M.); a-oonishi@vet.ous.ac.jp (A.O.); m-miyabe@vet.ous.ac.jp (M.M.); y-shimizu@vet.ous.ac.jp (Y.S.); k-saeki@vet.ous.ac.jp (K.S.); y-okamura@vet.ous.ac.jp (Y.O.); k-yamazoe@vet.ous.ac.jp (K.Y.)

**Keywords:** dysuria, hyperplasia, os penis, Pug dog, urethral obstruction

## Abstract

A three-year-old male Pug presented with a three-year history of urolithiasis and repeated urethral obstruction. Biochemical analysis, ultrasonography, and retrograde urethrocystography revealed probable portosystemic shunt and incomplete urethral obstruction due to uric acid ammonium calculi. Enhanced computed tomography (CT) revealed portosystemic shunt and proliferation of the osseous tissue of the os penis, which was surgically removed. Histopathologically, the excised osseous tissue comprised bland lamellar bone without atypia or inflammation. Hyperplasia of the os penis was diagnosed based on the image findings and histopathology. The dysuria improved postoperatively. This is the first report of dysuria associated with non-neoplastic bone hyperplasia of the os penis in a dog. Careful evaluation of the os penis by CT is needed for accurate diagnosis in case of repeated penile urethral obstruction.

## 1. Introduction

Dysuria is a common symptom of urinary system diseases in dogs. Dysuria is caused by lower urinary tract diseases, such as urolithiasis, urethritis, cystitis, and occupying mass lesion [1]. Complete or incomplete urethral obstruction is one of the main causes of dysuria, which is associated with urethral calculi or neoplasia in the urethra and os penis. There are some reports of urethral obstruction due to various tumors of the os penis [2,3,4,5]. Urethral obstruction due to neoplastic bone hyperplasia of the os penis with osteochondrosarcoma and osteosarcoma has been reported in dogs [4,5]. However, non-neoplastic bone hyperplasia of the os penis associated with dysuria has not been reported.

This report describes the computed tomography (CT) findings and detailed surgical management of an unusual case of dysuria associated with non-neoplastic bone hyperplasia of the os penis.

## 2. Case Presentation

This report was conducted according to the ethical guidelines of the Japan Veterinary Medical Association. Owner provided informed consent for all the procedures before these were undertaken.

A three-year old intact male Pug, weighing 13.1 kg, was referred to Okayama University of Science Veterinary Medical Teaching Hospital for a three-year history of urolithiasis with uric acid ammonium and repeated urethral obstruction.

**Figure 1:**
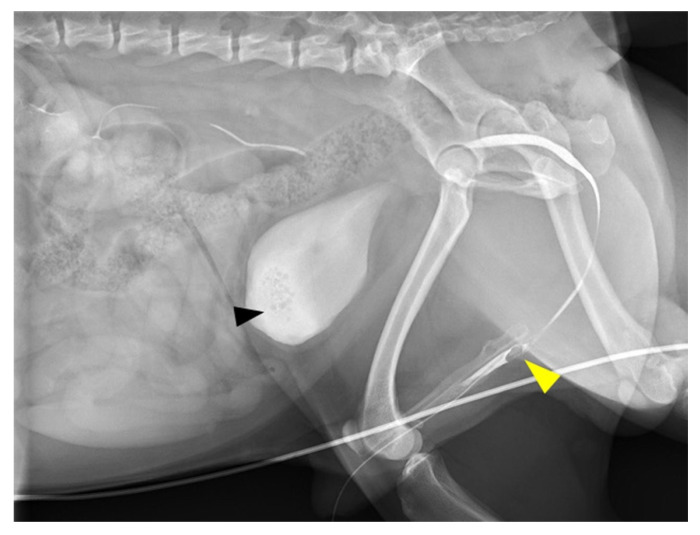
Retrograde urethrocystography image. Retrograde urethrocystography revealed small calculi in the urinary bladder (black arrowhead), and loss of the contrast medium at the base of the os penis (yellow arrowhead).

Dysuria was the only complaint at initial consultation. No abnormality was detected on physical examination. Complete blood count (CBC) revealed slight leukocytosis (204 × 10^2^/μL; reference range, 60–170 × 10^2^/μL), and biochemical analysis showed postprandial mild hyperammonemia (fasting: 33.8 mg/dL, postprandial: 84.0 mg/dL; reference range, 16.0–75.0 mg/dL) and high total bile acid concentration (fasting: 19.3 mg/dL, postprandial: 90.3 mg/dL; reference range, 0–5.0 mg/dL). On survey radiography, many small calculi were seen in the urinary bladder. No calculi were seen in the urethra. Radiography and ultrasonography revealed microhepatia and hypoplasia of the hepatic portal vein. The insertion of the urethral catheter was relatively difficult, but a 4-Fr catheter was inserted after several attempts. Urinalysis revealed ammonium urate crystals. Retrograde urethrocystography revealed many small calculi in the urinary bladder and loss of the contrast medium at the base of the os penis (Figure 1). A portosystemic shunt and incomplete urethral obstruction due to the uric acid ammonium calculus were suspected.

CT and CT angiography were performed under general anesthesia with a 16-slice multi slice CT scanner (Aquilion™ Lightning, Canon Medical Systems, Otawara, Japan). The scanning parameters were as follows: rotation time 0.75 s; slice thickness 1.0 mm; table speed 16 mm/rotation; X-ray tube voltage 120 kV; and X-ray tube current 100 mA. In CT angiography, iopamidol (Oypalomin 300, Fuji Pharma Co., Tokyo, Japan) was used as a contrast medium and was administered at a dose of 2.5 mL/kg (750 mg iodine/kg) via the cephalic vein with a power injector (Smart Shot, Nemoto Kyorindo, Tokyo, Japan). The injection time was fixed at 15 s.

**Figure 2:**
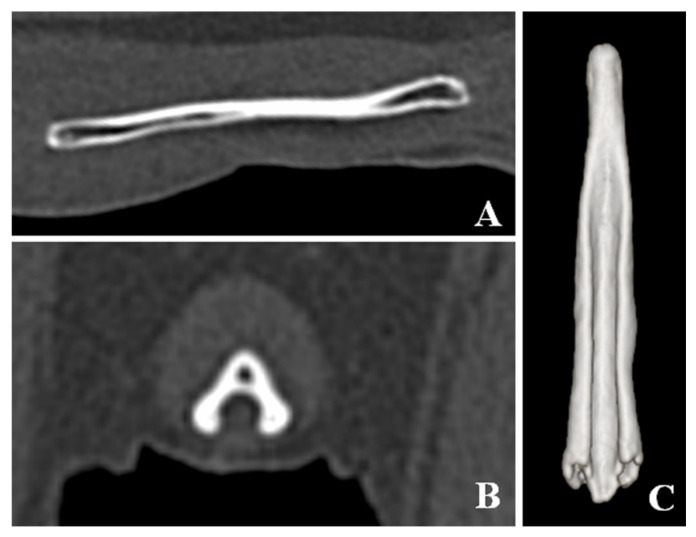
Computed tomographic images of the os penis in a normal dog. (**A**) Sagittal plane of the os penis. (**B**) Transverse plane of the os penis. (**C**) Ventral view of three-dimensional image of the os penis. The normal os penis is formed only by the urethral groove. The surface of the urethral groove is smooth.

**Figure 3:**
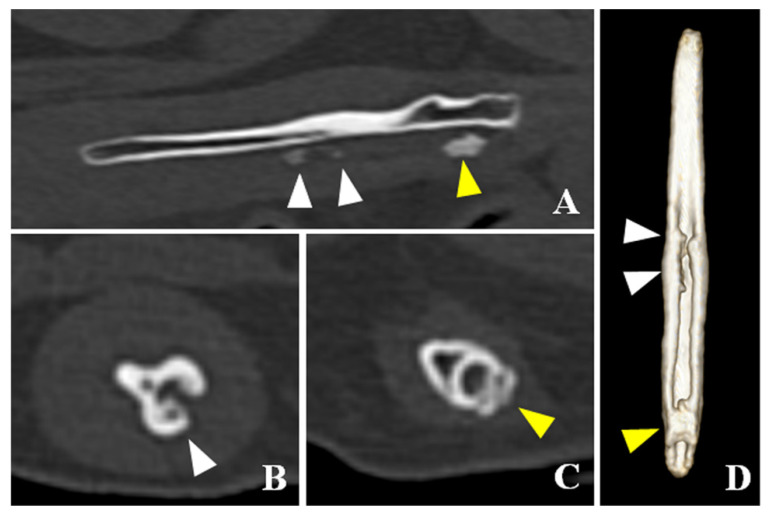
Computed tomographic images of the os penis in this case. (**A**) Sagittal plane of the os penis. (**B**) Transverse plane of the central portion of the os penis. (**C**) Transverse plane of the caudal portion of the os penis. (**D**) Ventral view of three-dimensional image of the os penis. In the caudal portion of the os penis, the arch of the osteoid tissues was formed surrounding the urethra (yellow arrowheads). In the central portion of the os penis, the surface of the urethral groove was nodulous due to the proliferation of the osteoid tissue (white arrowheads) but the arch was not Figure 4. Fr catheter confirmed the existence of solid osseous tissue at the base of the os penis, which displaced the urethra dorsally, causing incomplete occlusion of the urethral lumen. The solid osseous tissue was removed by rongeur and ultrasonic disintegrator (SonoCure ™, TOKYO IKEN Co., Ltd, Tokyo, Japan). After its removal, a 6-Fr catheter could pass through this area without any difficulty. Since the 6-Fr catheter could be inserted, the osseous tissue in the middle part of the penis was not immediately removed. The 6-Fr catheter was then placed there for ten days.

CT detected bladder calculi, urethritis, and proliferation of osseous tissue of the os penis. There was no calculus in the urethra. The normal ureteral groove of the dog was smooth (Figure 2); however, an arch of osseous tissues was formed around the urethra in the caudal portion of the baculum in this case (Figure 3A,C,D). The surface of the urethral groove was nodulous, with proliferation of osseous tissue (Figure 3A,B,D) in the central portion of the os penis. The arch was not formed in this region. In CT angiographic images, portosystemic shunt (left gastro-azygos shunt) was detected.

**Figure 4:**
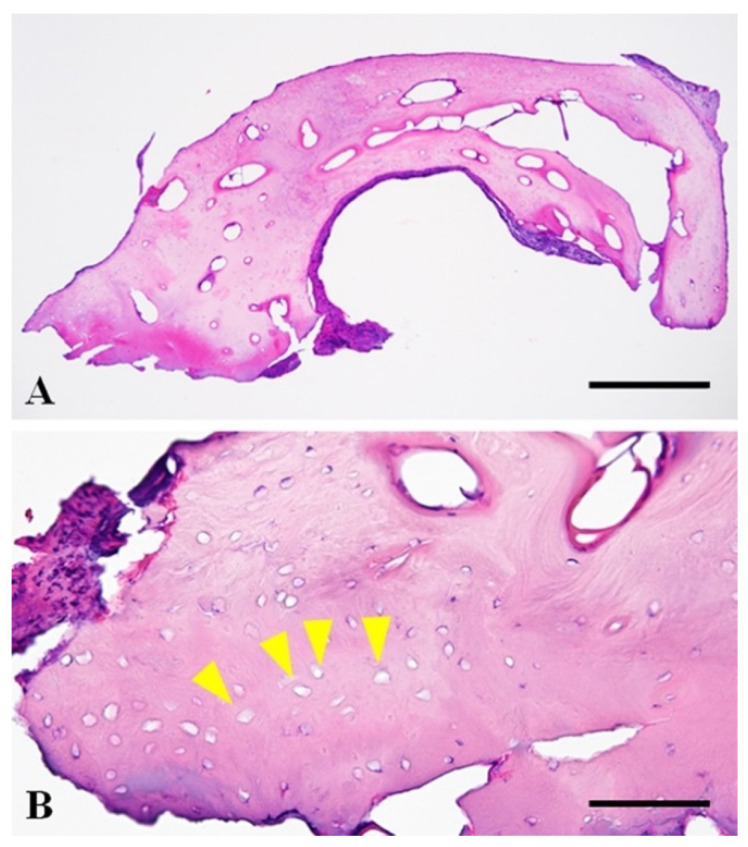
Photomicrographs of the resected osseous tissue from the os penis. (**A**) Photomicrograph ×40. The tissue was well-organized mature compact bone without neoplastic or inflammatory changes. HE. Bar = 500 µm. (**B**) Photomicrograph, 400×. The tissue was composed of evenly spaced bony lamellae around central canals. Most bony lacunae lack osteocytes, suggestive of necrosis (yellow arrowhead). HE. Bar = 100 µm.

The resected osseous tissue was submitted for histopathological examination. The tissue sections were routinely prepared after formalin fixation and a three-day decalcification with 5% formic acid. The tissue was composed of evenly spaced bony lamellae around the central canals, consistent with mature compact bone. Bony lacunae often lack osteocytes within them, which is suggestive of necrosis of the bony tissue. There was no neoplastic or inflammatory change in the submitted tissue (Figure 4). Dysuria associated with non-neoplastic bone hyperplasia was diagnosed based on these findings and clinical information.

The main components of the extracted calculus from the bladder were uric acid ammonium, calcium oxalate and magnesium ammonium phosphate (uric acid ammonium: 69%, calcium oxalate: 21%, magnesium ammonium phosphate: 10%).

The urethral catheter was removed 10 days after the surgery. The urinalysis showed a few red blood cells and white blood cells, but no crystals were found. Biochemical analysis revealed normal ammonia (fasting: 29.3 mg/dL, postprandial: 18.8 mg/dL; reference range, 16.0–75.0 mg/dL) and normal total bile acid concentrations (fasting: 0.8 mg/dL, postprandial: 2.4 mg/dL; reference range, 0–5.0 mg/dL). There was no difficulty in urination immediately after the withdrawal of the urethral catheter, but dysuria was occasionally noted for two months despite insertion of a 6-Fr catheter. Following that, the dysuria improved, and no difficulty in urination was observed for three months.

## 3. Discussion

The present dog presented with dysuria (urinary stasis in the urethra), despite the absence of calculus in the urethra. The urethra was narrowed at the point of the hyperplastic osseous tissue. Dysuria improved after removal of the bone that had narrowed the urethra and urethral catheter retention for 10 days. There are two possible causes for dysuria in this dog. This first possible cause is hyperplasia of the os penis or ectopic ossification of the tissue around the urethra due to chronic urethritis associated with repeated obstruction by uric acid ammonium calculi. The resected osseous tissue revealed non-neoplastic mature bone and necrosis of the bony tissue without inflammation. Inflammation was not detected in the hyperplastic osseous tissue, but inflammation or focal vascular insufficiency might have occurred at the urethra and the tissue surrounding the osseous tissue since necrosis of the bony tissue was detected. An ectopic ossification is the production of bone in an abnormal position that can occur in any tissue. A widespread ectopic ossification of soft tissue occurs in association with trauma [6]. The lesion has a characteristic sequence of maturation in which fibroblast-like cells differentiate and produce osteoid, which is further mineralized and ultimately replaced by lamellar bone [6]. The present patient had a suspected urethritis at the base of the os penis, but the resected osseous tissue did not show histopathologic findings of ectopic ossification.

Fracture of the os penis has been reported in a dog, which presented as urethral obstruction two years later [7]. The urethral obstruction in the dog was associated with callus and fibrous tissue proliferation, compressing the urethra at the fracture site. Proliferation of the bone was not found in the case of fracture [7]. The present case did not have a history of fracture of the os penis and did not show callus, fibrosis tissue, and signs of bone fracture on CT imaging.

The second possible cause is congenital malformation of the os penis. Repeated urethral obstruction might be caused by malformation of the os penis, which narrows the urethra. Urethral obstruction repeatedly occurred due to a narrowed urethra owing to the malformed os penis and portosystemic shunt. Finally, dysuria occurred due to chronic urethritis and secondary hyperplasia of the urethral tissue at the point of obstruction. A malformed os penis has been described to easily cause urethral obstruction in dogs [8]. However, while previous reports have mentioned the length and bending of the os penis, there was no mention of hyperplasia. However, hyperplasia of the os penis in this case may be a congenital malformation and a cause of dysuria. 

The bone hyperplasia of the os penis was revealed by CT imaging. In this case, the hyperplasia of the bone at the base of the os penis looked similar to urethral calculus in radiography and retrograde urethrocystography. It was difficult to capture the true three-dimensional form of the bone by radiography only. CT is recommended when urethral calculus is suspected in the penile part of the urethra.

## 4. Conclusions

Our report showed non-neoplastic bone hyperplasia of the os penis and associated dysuria in a dog. This non-neoplastic bone hyperplasia might be caused by repeated urethral obstruction by uric acid ammonium calculi in the portosystemic shunt or malformation of the os penis. From a three-dimensional point of view, CT is advised to evaluate the abnormality of the os penis in cases where urethral obstruction in the penile part of the urethra is suspected.

## Data Availability

Data sharing not applicable.
